# ESR Essentials: a step-by-step guide of segmentation for radiologists—practice recommendations by the European Society of Medical Imaging Informatics

**DOI:** 10.1007/s00330-025-11621-1

**Published:** 2025-05-22

**Authors:** Kalina Chupetlovska, Tugba Akinci D’Antonoli, Zuhir Bodalal, Mohamed A. Abdelatty, Hendrik Erenstein, João Santinha, Merel Huisman, Jacob J. Visser, Stefano Trebeschi, Kevin B. W. Groot Lipman

**Affiliations:** 1 Department of Radiology, Netherlands Cancer Institute, Amsterdam, The Netherlands; 2 GROW School for Oncology and Reproduction, Maastricht University, Maastricht, The Netherlands; 3 Department of Diagnostic and Interventional Neuroradiology, University Hospital Basel, Basel, Switzerland; 4 Department of Pediatric Radiology, University Children’s Hospital Basel, Basel, Switzerland; 5 London North West University Healthcare NHS Trust, London, UK; 6 Department of Medical Imaging and Radiation Therapy, Hanze University of Applied Sciences, Groningen, The Netherlands; 7 Department of Radiotherapy, University of Groningen, University Medical Centre Groningen, Groningen, The Netherlands; 8 Research Group Healthy Ageing, Allied Health Care and Nursing, The Hanze University of Applied Sciences, Groningen, The Netherlands; 9 Digital Surgery LAB, Champalimaud Research, Champalimaud Foundation, Lisbon, Portugal; 10 Instituto Superior Técnico, Universidade de Lisboa, Lisbon, Portugal; 11 Department of Radiology and Nuclear Medicine, Radboud University Medical Center, Nijmegen, The Netherlands; 12 Department of Radiology & Nuclear Medicine, Erasmus University Medical Center, Rotterdam, The Netherlands; 13 Department of Thoracic Oncology, Netherlands Cancer Institute, Amsterdam, The Netherlands

**Keywords:** Segmentation, Volumetry, Imaging, Artificial intelligence

## Abstract

**Abstract:**

High-quality segmentation is important for AI-driven radiological research and clinical practice, with the potential to play an even more prominent role in the future. As medical imaging advances, accurately segmenting anatomical and pathological structures is increasingly used to obtain quantitative data and valuable insights. Segmentation and volumetric analysis could enable more precise diagnosis, treatment planning, and patient monitoring. These guidelines aim to improve segmentation accuracy and consistency, allowing for better decision-making in both research and clinical environments. Practical advice on planning and organization is provided, focusing on quality, precision, and communication among clinical teams. Additionally, tips and strategies for improving segmentation practices in radiology and radiation oncology are discussed, as are potential pitfalls to avoid.

**Key Points:**

- *As AI continues to advance, volumetry will become more integrated into clinical practice, making it essential for radiologists to stay informed about its applications in diagnosis and treatment planning*.
- *There is a significant lack of practical guidelines and resources tailored specifically for radiologists on technical topics like segmentation and volumetric analysis*.
- *Establishing clear rules and best practices for segmentation can streamline volumetric assessment in clinical settings, making it easier to manage and leading to more accurate decision-making for patient care*.

## Key recommendations

- A protocol should be planned in advance to ensure segmentations address clinical questions and maintain consistency through incorporating standards for image quality, suitable slice thickness, and selected planes, phases, or sequences (level of evidence: moderate).
- Adherence to the digital imaging and communications in medicine (DICOM) standard, specifically using the DICOM SEG object for radiological segmentations (level of evidence: moderate).
- In the case of AI-assisted segmentation, a quality control framework should be employed to track the performance on segmentation performance, e.g., dice similarity coefficient (DSC), as well as clinical workflow performance, e.g., radiologist adjustment time (level of evidence: high).

## Introduction

Anatomical structures are inherently three-dimensional, but in routine clinical practice, measurements are often simplified to short and/or long-axis diameters due to the challenges of obtaining total volume. However, with advances in AI, volumetric measurements are becoming more achievable through image segmentation. Traditionally, a labor-intensive and time-consuming task when done manually, segmentation is now increasingly automated or semi-automated, making it more accessible and efficient. During segmentation, each voxel in the scan is classified as part of a specific structure or area of interest or as background. When done manually, often through image delineation or contouring (a technique long established in radiotherapy planning for target volumes and organs-at-risk [1]), the region’s border is outlined, and all voxels within this boundary are assigned the same label. This data can be used to train machine learning models while enabling cohort analyses such as treatment response evaluation and radiomics analysis [2]. In clinical practice, segmentation can support accurate lesion detection and longitudinal tracking, facilitate volumetric measurements and growth rate assessments of tumors for disease monitoring, and assist in planning for surgeries and radiation therapy. Furthermore, segmentation can help to extract valuable body composition data and be useful in measuring bone density for osteoporosis, visceral fat for metabolic syndrome, and muscle mass for sarcopenia and muscle dystrophies [3].

**Fig. 1:**
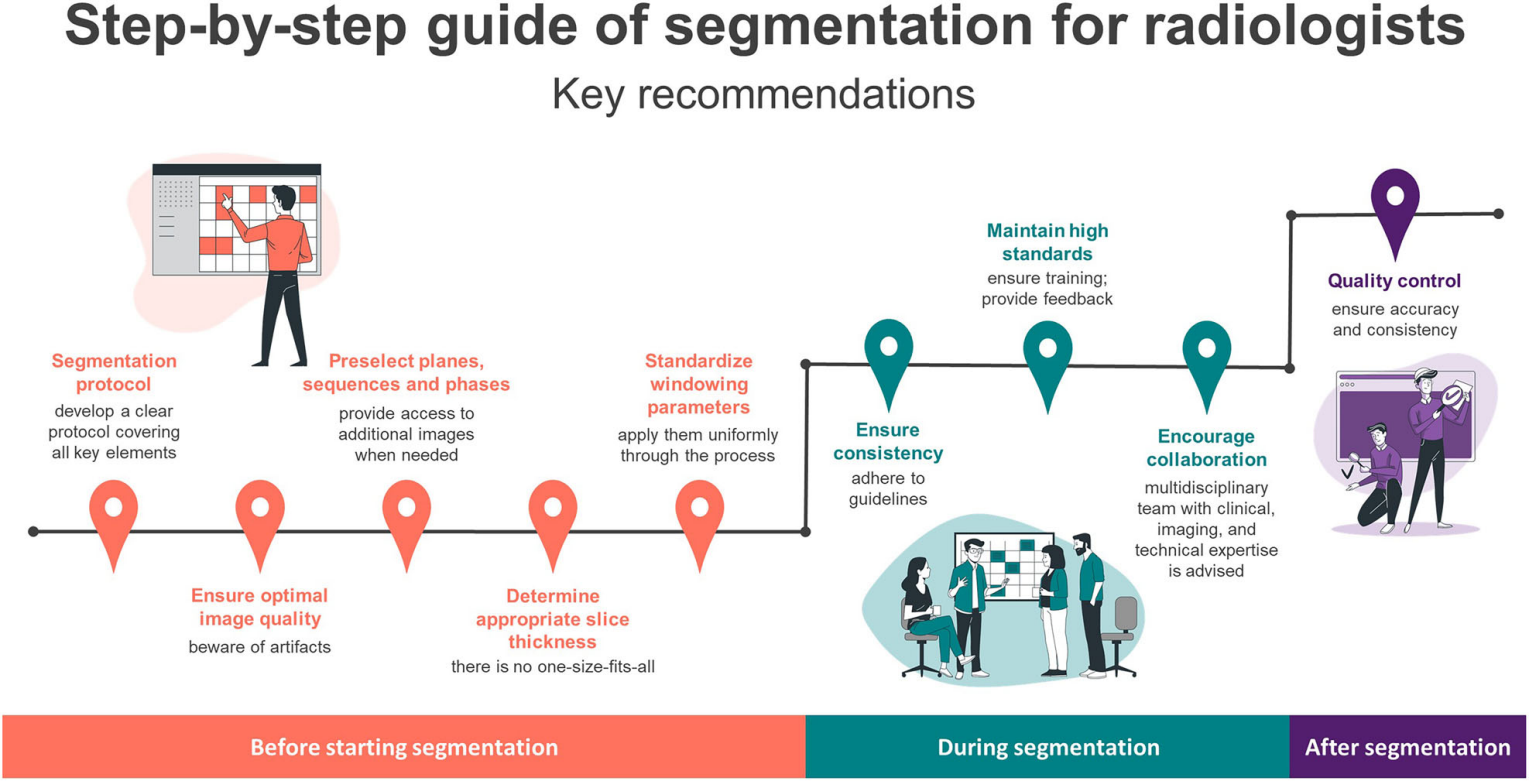
A chart outlining the main recommendations for performing segmentations. Figure 1 was created with Storyset (https://storyset.com)

Radiologists, whether in research or clinical settings, may be required to participate in generating, reviewing, or evaluating segmentations. While we will refer mainly to radiologists in this paper, segmentations can be performed by multiple other professionals who interact with the images, such as radiotherapists, medical students, residents, physicists, and radiographers. To better support those performing this task, there is a need for clearer, more tailored guidelines. We present recommendations developed through the combined expertise of radiologists, computer engineers from a multidisciplinary research lab, and key EuSoMII members. These guidelines are specifically crafted to address non-technical aspects and common challenges encountered in segmentation. Our aim is to provide detailed, step-by-step instructions and practical advice to help radiologists and imaging professionals handle segmentation more effectively (Fig. 1).

## Key recommendations

### Developing a segmentation protocol

The setup of segmentation protocols in clinical and research settings requires standardization, where input from both medical and technical experts is necessary for protocol development. Here, running initial test cases helps to refine the process and spot potential issues, which creates an iterative improvement cycle. The final protocol requires several key elements: clear goals, patient selection guidelines, imaging standards, and chosen software tools (such as 3D Slicer [4] or ITK-Snap [5]). Adding quality checks, clear data management steps, detailed documentation guidelines, and evaluation methods creates a complete framework that works well for both clinical use and research needs [6]. In radiotherapy, studies have shown that implementing a clear segmentation protocol can reduce the clinical target volume and interreader variability [7]. Additionally, supplementing a written protocol with a visual atlas, consensus treatment guidelines, and structured training sessions has been demonstrated to improve clinical target volume delineation accuracy, bringing it closer to expert-derived contours while improving consistency across participants [8, 9].

Standardized checklists can offer valuable guidance for developing and evaluating segmentation protocols. For example, RIDGE emphasizes reproducibility in segmentation models [10], CLAIM promotes transparent reporting of AI-driven segmentation studies [11], and CLEAR supports quality assessment in radiomics research [12].

### Ensuring optimal image quality

High-quality images help radiologists generate accurate segmentations more efficiently. Generally, if the image quality is not suitable for clinical use, it is likely not adequate for performing segmentation and volumetric analysis either.

Images with low signal-to-noise ratios (SNRs) might be suboptimal for voxel-level tasks like segmentation, particularly in CT images when employing sharp kernel reconstructions for soft tissue. Soft tissue reconstructions are recommended for segmenting soft tissue structures or lesions in CT, as the increased noise associated with sharp reconstructions can obscure the fine details necessary for precise segmentation. Several objective methods can be used to evaluate medical image quality. SNR measures the strength of the true signal relative to background noise, while contrast-to-noise ratio (CNR) assesses how well a structure of interest stands out from its surroundings. The Structural Similarity Index compares an image to a reference to detect distortions and assess overall image fidelity. These metrics provide quantifiable and reproducible assessments that complement expert assessments [13].

Artifacts, including those caused by breathing or movement, CT beam-hardening, MRI signal loss, or inadequate fat suppression, can occur in clinical settings and may impact results [14]. To address these challenges, the segmentation interface can include options for users to assess and document artifacts in a structured manner, as image quality preferences may vary according to the specific task [15]. For instance, implementing a Likert scale rating for the severity of artifacts facilitates the investigation of their potential impact on segmentation accuracy. Exclusion of images should only be considered in cases where artifacts severely compromise their quality and reliability, rendering them unusable for segmentation. AI-based models should be capable of handling suboptimal image quality while still maintaining reasonable performance. Developing robust and generalizable models requires addressing the real-world variations encountered in clinical practice, including artifacts and imperfections, to ensure consistent, reliable segmentation across diverse scenarios and imaging conditions.

### Determining appropriate slice thickness

**Fig. 2:**
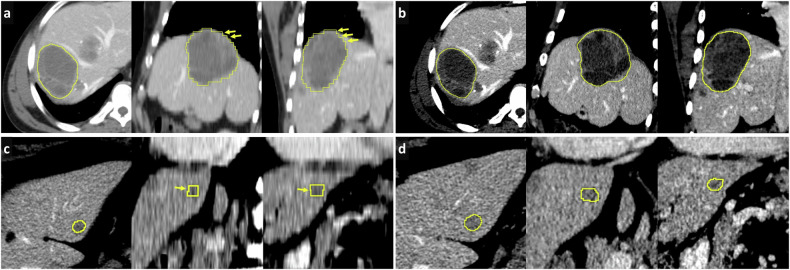
CT images showing liver metastasis segmentation performed on the axial plane in 5-mm slice thickness (**a**) vs 1-mm slice thickness (**b**), displayed in axial, sagittal, and coronal views. Thicker slices yield a volume of 175 cm³ compared to 167 cm³ for thinner slices—a 4.5% difference. The difference is even more noticeable in a smaller lesion, with volumes of 0.97 cm³ at 5 mm (**c**) compared to 0.85 cm³ at 1 mm—a 12.3% difference (**d**). Note the stair-step appearance (arrows) and reduced detail in the coronal and sagittal views for the thicker slices

Slice thickness may impact segmentation quality [16], inter-observer variability [17] and the reproducibility of radiomics features [18, 19], as well as affect the segmentation volume (Fig. 2). Ensure consistency in slice thickness for all segmentations, either by including only scans with that specific thickness, or by down-sampling the scans (e.g., 1.2 mm or 1.5 mm can be converted to 3 mm) together with technical colleagues. Upsampling (e.g., converting a 5 mm to a 3 mm) is suboptimal, as interpolation may introduce fictitious information. Thinner slices are more sensitive to noise and artifacts and may increase segmentation time, especially for larger structures. However, they allow for more detailed segmentation and thus a more representative volume. Thicker slices allow for faster segmentation but are more prone to partial volume effects and may result in loss of information [19, 20]. Optimal slice thickness is structure-dependent, and there is no one-size-fits-all; it depends on the imaging modality and the structures that need to be segmented. For example, brain metastases on MRI may require thinner slices than segmenting liver lesions on CT. As a general rule, we recommend using the slice thickness used in MPR mode for clinical reporting on the structure. Literature suggests that organ volumetry can tolerate thicker slices, typically between 3 mm and 5 mm, as small variations in segmentation have minimal impact on overall volume calculations [21]. Body composition analysis can allow even thicker slices (≥ 5 mm) since fat, muscle, and bone density measurements rely more on overall tissue distribution than fine structural details [22, 23]. In contrast, radiomics analysis may benefit from thinner slices (≤ 2 mm), as finer resolution leads to the extraction of more robust and reproducible features [24–26]. Importantly, the time radiologists can dedicate also plays a crucial role, as segmentation is a labor-intensive and time-consuming task [27]. Fully manual segmentation performed by radiologists can take anywhere between a few and 150 min per single lesion, depending on the size of the lesion and the complexity of the case [28–33]. A reasonable compromise to balance accuracy and efficiency is key.

### Preselecting planes, sequences, and phases for consistent segmentation

The plane in which a segmentation is performed can significantly affect its shape and form. Thus, maintaining consistent segmentation planes is recommended. Typically, segmentations are performed along the acquisition plane, usually the axial plane, which offers higher resolution. Another plane might be selected in rare scenarios, especially when working with MRIs. Communication on such a choice is critical, and consistency is paramount.

**Fig. 3:**
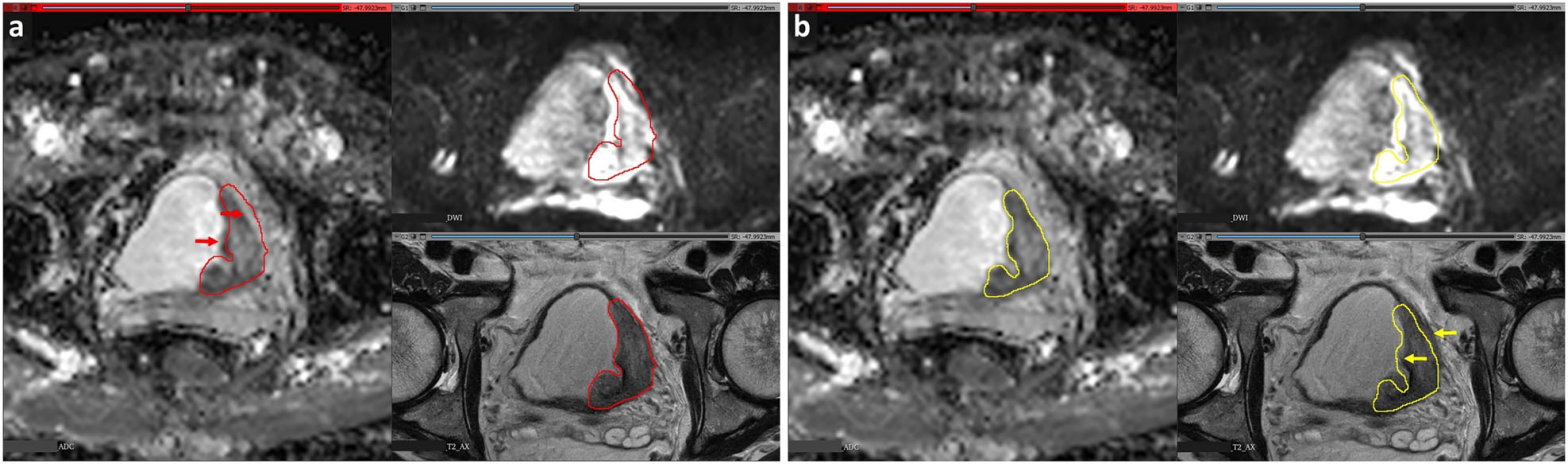
Axial MRI images using a 3D Slicer interface designed for segmentations of bladder cancer that allows for the simultaneous display of multiple sequences (ADC, DWI, and T2W). Comparison is presented between a segmentation performed on T2W images (**a**) vs one performed on ADC images (**b**). Notice the differences in border alignment and shape as these segmentations are being transposed on the other sequences

For MR images, sequences for annotation should be selected in advance, depending on the sequences most universally available across the patient cohort, and most importantly, those most clinically relevant with the best quality. Segmentations performed on one sequence are not immediately transferable to another sequence with different parameters; some level of image alignment is likely necessary. Image registration, the process of aligning medical images from different time points, modalities, or sequences, can help mitigate these differences by ensuring accurate comparisons and analyses [34]. However, even after alignment, the borders of the structure segmented on different sequences might differ (Fig. 3). Accordingly, if segmentation of multiple sequences is needed, even after successful registration, the radiologist might still have to perform additional corrections or adjustments to account for residual differences in structure boundaries across sequences. To reduce the workload, it is best to keep the number of sequences to a reasonable minimum. However, it is important to note that even if segmentation is performed on a single sequence, providing additional sequences for reference can improve accuracy and confidence. For example, when segmenting rectal cancers on axial T2W images, radiologists may find it helpful to also review sagittal and coronal T2W, DWI, and ADC images. This can be achieved by creating an interface (e.g., in Fig. 3, with 3D Slicer) that simultaneously displays all necessary sequences or by ensuring radiologists have access to the full set of images stored on a picture archiving and communication system or elsewhere to view them as needed. The same applies to contrast phases, as radiologists in clinical practice usually refer to all contrast phase images to identify a lesion’s enhancement pattern [35].

**Fig. 4:**
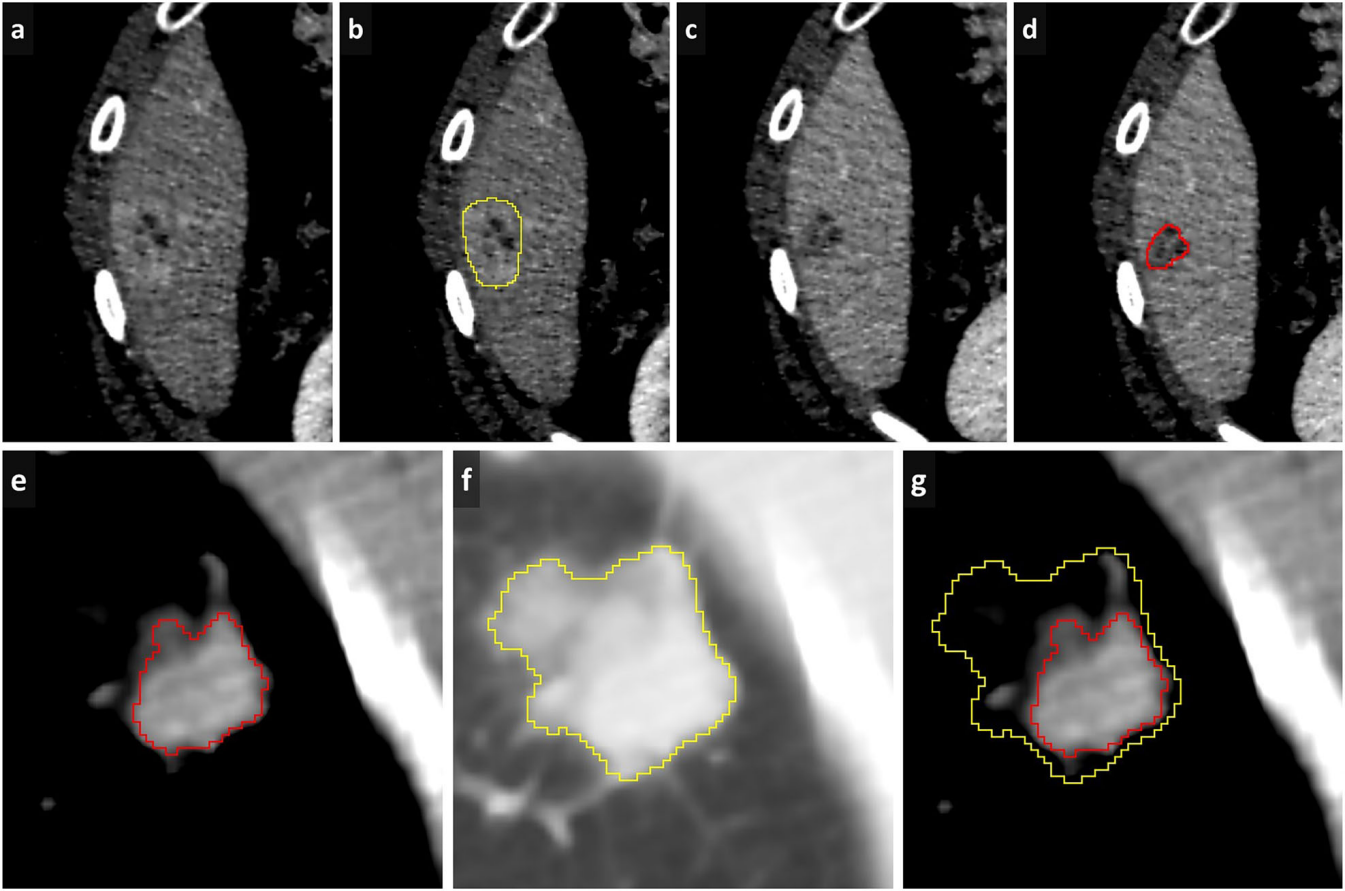
**a**–**d** Axial CT images of a liver lesion (neuroendocrine tumor metastasis) in the arterial (**a**, **b**) and portovenous (**c**, **d**) phases, showing differences in segmentation size and shape, with calculated volumes of 6.7 cm³ and 3.0 cm³, representing a 55% variation. **e**–**g** Segmentations of a lung lesion using soft tissue (**e**) and lung (**f**) windows, with a comparison of both segmentations shown in (**g**). Volume calculations reveal a significant difference—4.7 cm³ vs 10.8 cm³—resulting in a 56% variation

When using contrast-enhanced images for segmentations, consistency in the selected phase is essential, where the one that best visualizes the target structures or lesions should be selected. The contrast phase can significantly affect a lesion’s appearance, including its size, shape, borders, density/intensity, and internal heterogeneity (Fig. 4). In radiation oncology, it has been shown that contrast phase selection may significantly influence target delineation [36, 37]. Using images from the same contrast phase helps ensure that any differences observed between exams or patients are due to actual differences in the tissue, rather than variations inherent to different phases. Phase information should be included in the DICOM tags. If it is unavailable and the preferred phase cannot be easily pre-selected before segmentation, consider conducting a preliminary random cohort selection to estimate phase frequency or have radiologists indicate the phase during segmentation.

### Standardizing windowing parameters

In radiation oncology, studies have shown that window settings influence segmentation accuracy, with appropriate window level selection improving treatment volume delineation and reducing unnecessary radiation exposure [38, 39]. Selecting a specific windowing setting and applying it uniformly throughout the segmentation process helps maintain consistency. Even minor changes in window settings can lead to discrepancies in segmentation dimensions and shapes across slices, studies, and readers. For instance, using a liver-specific window (e.g., window width: 200 and window level: 100) can enhance the visibility of liver lesions compared to a standard abdominal window. Window selection is particularly crucial for lung lesions, where significant variations in size and shape can occur between segmentations performed on lung vs soft tissue windows (Fig. 4). In fact, some lesions with semi-solid or ground-glass density might not even be visible on a soft tissue window [40]. In clinics, lung nodules are usually measured in the lung window on lung reconstruction CTs as per the recommendations of the Fleischner Society [41].

### Ensuring consistency in segmentations

Dividing the segmentation task among multiple segmenters reduces the workload on each person but results in a higher risk of inconsistency issues [42]. If multiple readers are working on the project, it is important to ensure they are all trained for the segmentation task to avoid a learning effect and minimize variability as much as possible. Moreover, employing a review process with consensus may help reduce interobserver variability [43]. Assessing interobserver variability with a set of training cases further enhances consistency by identifying discrepancies in segmentation approaches. Power analysis can help determine the optimal number of training cases by considering variability, effect size, and confidence level, ensuring statistical validity and reliability in segmentation studies. It is important to note that acceptable levels of interobserver variability depend on the clinical application and the anatomical structures being analyzed, with larger, well-defined organs generally showing higher agreement, while smaller or irregularly shaped lesions tend to have greater variability [44].

**Fig. 5:**
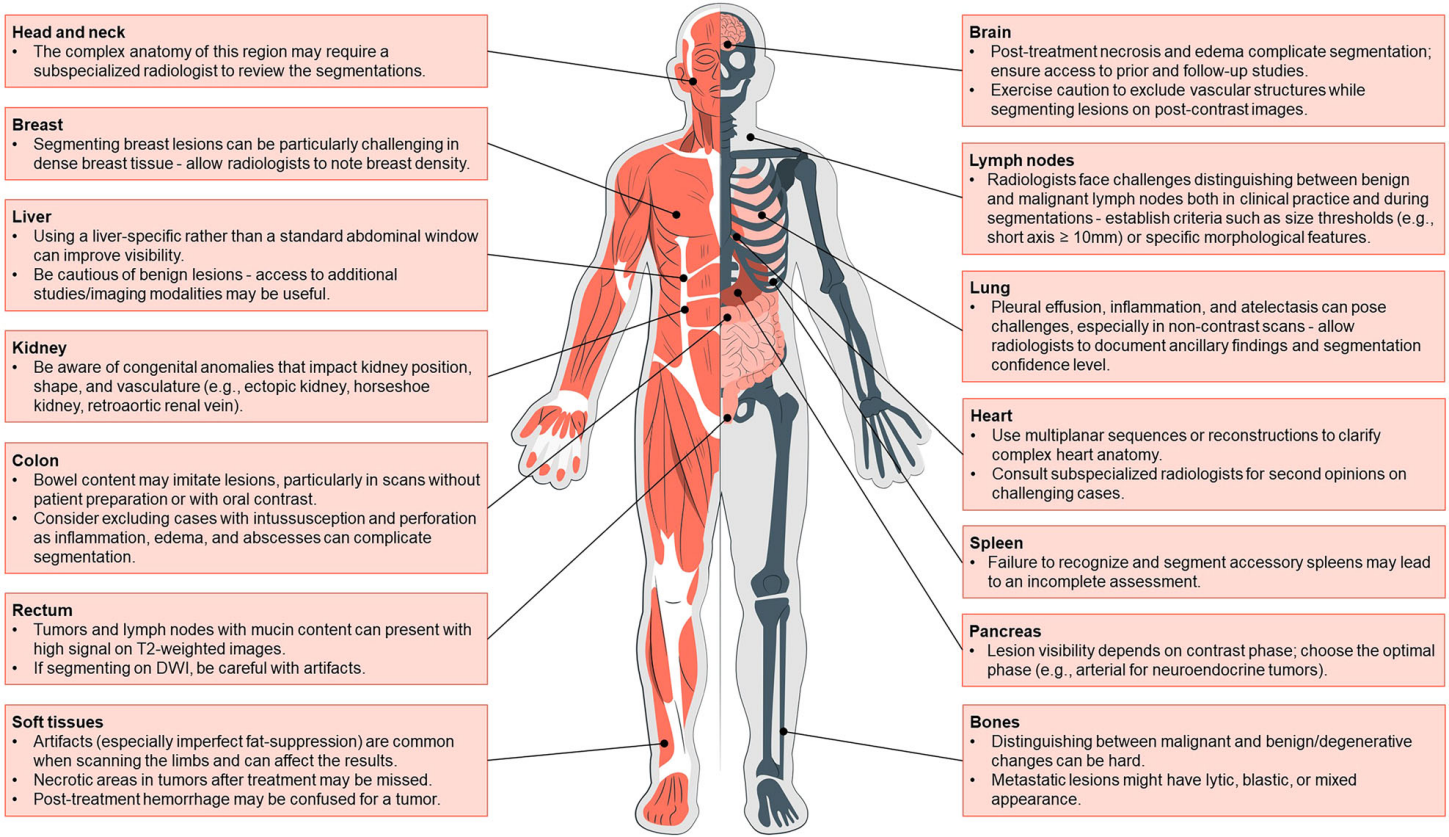
An illustration providing specific insights and potential challenges related to segmenting different body parts and types of lesions. Figure 5 was created with Storyset (https://storyset.com)

All segmenters should use the same software (and software version) and save files in standardized, reliable formats with clear naming conventions, ensuring data integrity and easy retrieval [45]. Automating the saving and documentation process as much as possible minimizes the risk of errors. Documentation of predefined choices can also include specific pearls and pitfalls related to the anatomical structure or lesion being segmented (Fig. 5). It’s important to stress the need for precise segmentations that exclude surrounding normal tissues or structures. This holds especially true if radiomics analyses are to be performed: the more precise the segmentation of the volume, the more stable and reproducible the extracted features [46, 47]. Inconsistent segmentation can cause significant variations in shape-based, texture-based, and intensity-derived feature measurements, potentially compromising radiomics model stability [48, 49]. Additionally, segmentation variability may hurt the performance of machine learning models, as extracted features might not accurately represent the tumor or tissue’s true biological characteristics.

**Fig. 6:**
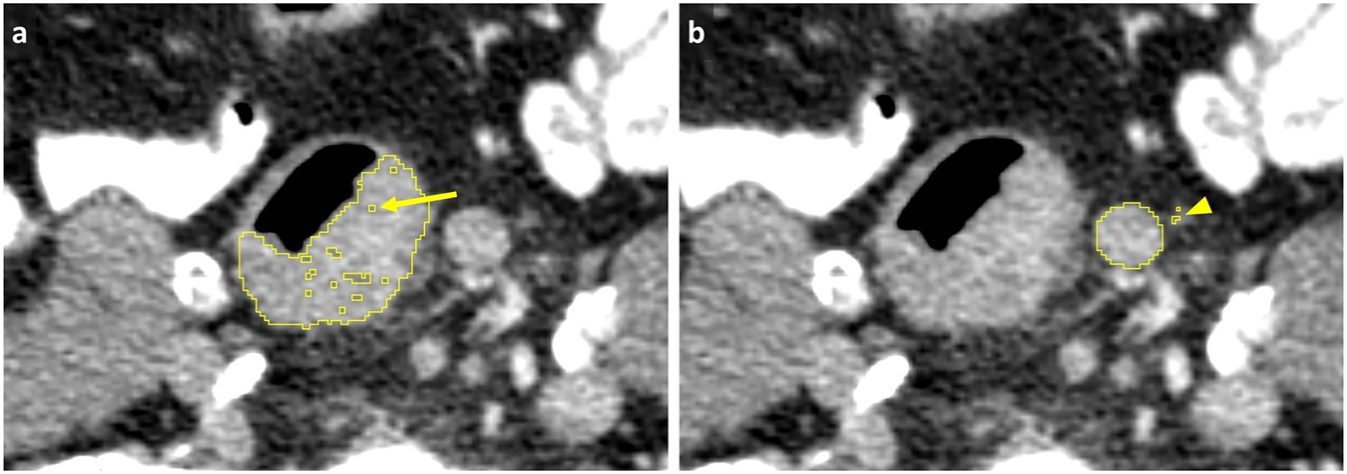
Axial CT images showing a segmentation of colon cancer (**a**), illustrating unsegmented pixels within the lesion (arrow), and a segmentation of a lymph node (**b**), highlighting “floating” pixels (arrowhead) adjacent to the main segmentation

It is of utmost importance that structures and lesions are thoroughly and consistently segmented across all slices. A common suboptimality that may be overlooked is leaving some pixels within the lesion unsegmented or segmenting small adjacent pixels not connected to the lesion (known as “floating pixels”), which barely affects volumetric measurement but can affect radiomics analysis (Fig. 6). Connected components analysis labels and identifies groups of connected pixels in a binary image based on their connectivity. By evaluating the size of each component and applying a size threshold, small components (floating voxels) can be removed to refine segmentations.

### Safeguarding against automation bias

Fully automatic segmentation through AI models could improve efficiency and potentially limit inter-observer variability. However, its accuracy can be compromised when encountering unfamiliar or complex data. AI-assisted segmentation serves as a middle ground, where AI outputs require human oversight and refinement to ensure reliability. A key concern here is automation bias, where users may over-rely on AI results. This is particularly dangerous in clinical work, as high workloads, time constraints, and task complexity increase the risk of automation bias, especially among inexperienced radiologists who are more likely to rely on incorrect AI-generated recommendations without verification [50]. To minimize these risks, it is important to balance automation with expert validation through human oversight, quality control measures, and ensuring AI-driven segmentation remains interpretable. A quality control framework should be employed to track the AI performance on both segmentation performance, e.g., DSC and normalized surface distance, as well as clinical workflow performance, e.g., radiologist adjustment time [51, 52]. If the DSC of the same AI model is increasing over time while the adjustment time is decreasing, automation bias could be at play.

### Maintaining high standards in segmentation quality

An experienced radiologist is not a guarantee for quality segmentations, and there could be not only inter-observer but also intra-observer variability [53, 54]. Besides correctly identifying the relevant anatomy and pathologies in medical imaging, segmentation requires precision and skills in using the specific software.

Providing radiologists with comprehensive clinical information, such as details about surgical or interventional procedures and treatments received, can help them to make informed decisions on challenging cases. Additionally, reviewing prior (or follow-up) studies or images from other imaging modalities can improve consistency, if available.

**Fig. 7:**
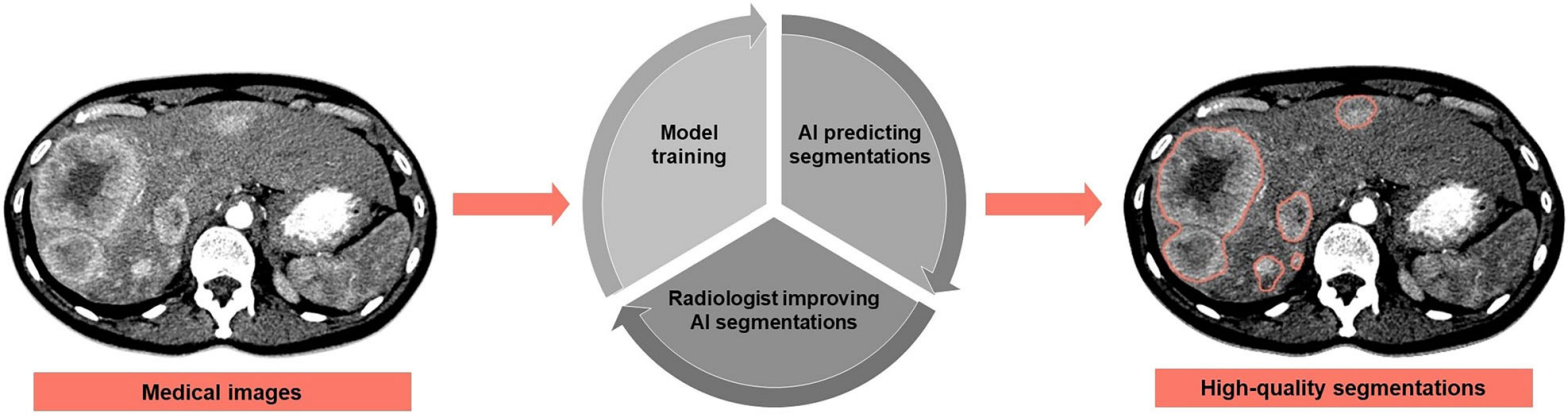
Illustration demonstrating the process where AI predicts segmentations that a radiologist then corrects. This cycle leads to the improvement of the algorithm, a decrease in the total amount of manual labor, and ultimately to high-quality automatic segmentations

Radiologists should recognize that providing accurate initial segmentations improves performance in research projects involving training an AI model. Models trained on high-quality data produce more precise automatic segmentations, reducing the need for manual corrections. Corrected segmentations can be fed back into the algorithm as high-quality training data, helping to further improve its accuracy and reliability. Over time, this iterative process of prediction, correction, and retraining enhances the model’s performance while gradually reducing the need for manual intervention (Fig. 7). Furthermore, radiologists should be aware that the quality and consistency of segmentations can significantly influence the quality of the resulting analysis [55]. For many organs and other tissues, tools like TotalSegmentator can provide accurate initial segmentations to reduce the workload [56, 57].

Constructive and timely feedback is essential for facilitating high-quality segmentations. Furthermore, each reader should complete a few training cases to help them gain proficiency with the tools or interfaces they will use. This initial practice helps them become comfortable with the system, reducing the impact of any learning curve on their segmentation accuracy during actual tasks. Moreover, less experienced segmenters can benefit from working through a series of test cases, which can then be reviewed and refined with input from seasoned colleagues.

Multiple metrics can be used to evaluate the quality of medical image segmentation, with the DSC, normalized surface distance, Jaccard Index, and Hausdorff Distance among the most commonly employed [52]. The Dice and Jaccard metrics assess the overlap between the predicted segmentation and the ground truth, providing a similarity measure, while the normalized surface distance and Hausdorff distance focus on boundary accuracy by measuring the distances between the edges of the segmented regions.

## Adherence to the DICOM standard

While researchers often use the NIfTI file format to develop AI segmentation models [58], segmentations produced in the clinical workflow should adhere to the DICOM standard [59]. The DICOM SEG object standard has voxel-level labels and is tied to the original DICOM series, avoiding mismatches between segmentation objects and corresponding images. Moreover, DICOM SEG objects allow changes, unlike DICOM secondary captures. The RT STRUCT format used in radiation oncology is geometry-based, which can lose the voxel-level labels. Therefore, DICOM SEG is recommended for radiologic applications [60, 61].

### Encouraging collaboration and communication

It is advisable to have a multidisciplinary team with clinical, imaging, and technical expertise [62]. Maintain open communication among radiologists, radiographers, radiotherapy specialists, and the team’s computer engineers or informatics specialists. Regular interactions between these roles allow for timely feedback and can streamline workflows and save time. For instance, computer engineers can develop custom extensions or interfaces specifically suited to the needs of radiology and radiotherapy teams, improving both functionality and usability.

## Summary statement

Medical image segmentation has the potential to improve the accuracy of measurements and is fundamental to advancing AI-based radiological research and diagnostics. High-quality segmentation requires careful planning, consistent parameters, and effective team communication. By adhering to best practices and guidelines, radiologists can ensure accurate, precise, and robust segmentations essential for consistent volumetric measurements.

## Patient statement

Segmentation is a technique that allows doctors to determine the volume of specific anatomical structures, such as tumors, with a high level of precision and accuracy. In this paper, we aimed to help imaging specialists enhance the accuracy and consistency of segmentation by setting clear protocols, standardizing the tools and methods used, and incorporating automation for specific steps. Making segmentation more reliable could help doctors provide a better overview of treatment effects, potentially leading to better patient outcomes and improved quality of care.

## Abbreviations

DICOM: Digital imaging and communications in medicine
DSC: Dice similarity coefficient
SNR: Signal-to-noise ratio
